# Outcomes of Patients with Monoclonal Gammopathy of Undetermined Significance with and without Atrial Fibrillation: A Retrospective Cohort Analysis of the Nationwide Inpatient Sample Database

**DOI:** 10.3390/jcm12134436

**Published:** 2023-06-30

**Authors:** Rachelle Hamadi, Zakaria Alameddine, Samer Asmar, Fouad Sakr, Hussam Aridi, Reem Dimachkie, Hadi Skouri

**Affiliations:** 1 Internal Medicine Department, Staten Island University Hospital, 475 Seaview Avenue, Staten Island, NY 10305, USA; rhamadi@northwell.edu (R.H.); sasmar1@northwell.edu (S.A.); fsakr@northwell.edu (F.S.); haridi@northwell.edu (H.A.); rdimachkie@northwell.edu (R.D.); 2 Cardiology Department, Sheikh Shakhbout Medical City-Mayo Clinic, Abu Dhabi P.O. Box 11001, United Arab Emirates; hskouri@ssmc.ae

**Keywords:** MGUS, AFIB, mortality, AKI, pericarditis

## Abstract

Background: Monoclonal gammopathy of undetermined significance (MGUS) is a non-malignant precursor of multiple myeloma (MM). MGUS has been suggested to be associated with a higher risk of cardiovascular diseases, including AFIB, but it is still unclear whether this association is real. Studies are lacking on the impact of atrial fibrillation on health outcomes in this population. The association of AFIB in this population is lagging and merits further investigation. Methods: The study conducted a retrospective analysis of the Nationwide Inpatient Sample (NIS) for 2018, including adult patients with primary diagnoses of MGUS and AFIB. Patients were divided into two groups based on AFIB presence. Outcomes assessed included complications, length of stay, mortality, hospital charges, and discharge disposition. Results: The study included 9007 patients with MGUS of whom 2404 had AFIB. Patients with both MGUS and AFIB had higher rates of acute kidney injury [AKI] (31.5% vs. 27.5%; *p* = 0.002) and pericarditis (2% vs. 1.2%; *p* = 0.029). They also had longer hospital stays (5 vs. 4 days; *p* < 0.001) and higher hospitalization costs ($43,729 vs. $41,169; *p* < 0.001). Conclusions: The study showed that the prevalence of AFIB in MGUS patients is high. Patients with AFIB had increased rates of complications (AKI and pericarditis) and higher mortality compared to patients without AFIB. Further studies screening for AFIB in this patient population are warranted.

## 1. Introduction

Multiple myeloma (MM) is a hematologic malignancy that is characterized by the clonal proliferation of plasma cells in the bone marrow. A precursor stage of MM is known as monoclonal gammopathy of undetermined significance (MGUS), which is a non-malignant condition where a monoclonal protein is detected in the blood but there is no evidence of disease [1]. MGUS is characterized by the presence of a monoclonal protein in the blood (<3 g/dL), bone marrow with less than 10% monoclonal plasma cells, and the absence of end-organ damage resulting from the proliferative process [2].

This condition has three clinical types, which include immunoglobulin M (IgM) MGUS, non-IgM MGUS, and light-chain MGUS, and that can progress to malignancy through different pathways [3]. Notably, the prevalence of MGUS in the general population increases with age, from around 0.3% in patients younger than 50 years to around 3% in those older than 50 years of age [3]. Although MGUS has traditionally been viewed as a benign precursor to malignancy, some studies suggest that it may be associated with an increased incidence of diseases independent of malignancy [2]. However, whether this association is real or coincidental is ongoing and cannot be determined without a study with a long follow-up period [4]. The presence of other comorbid conditions that may affect the development of cardiovascular risk factors makes it difficult to establish a causal relationship between MGUS and these diseases. Age is a major risk factor for the development of both MGUS and atrial fibrillation (AFIB). The prevalence of AFIB increases with age. It is estimated to be 1% worldwide, but it increases to 9% in patients over the age of 75 and to 22% for patients older than 80 years [5]. The percentage of clinically recognized cases among prevalent cases of MGUS is 13% at 60 years of age, but this increases to 33% by the age of 80 years [6].

Schwartz et al. performed a large database study where they compared MGUS patients to patients without MGUS to better understand the latter’s impact on the general population. The results of the study showed that MGUS was associated with an increased risk for cardiovascular disease, including heart failure, acute myocardial infarction, stroke, AFIB, and some valvular heart disease. Interestingly, they also conducted a sensitivity analysis where they only included patients without specific comorbidities (such as diabetes mellitus, hypertension, myocardial infarction, and chronic renal disease). The results of such analysis had similar outcomes which drove us to investigate the impact of AFIB on such a population [2].

Additionally, the incidence of atrial fibrillation in individuals with MGUS, the clinical implications, and in-hospital complications of patients diagnosed with both MGUS and AFIB has not been well studied in the past. The aim of this study is to compare the demographic, admission, and medical comorbidity characteristics of patients with MGUS with and without AFIB.

## 2. Materials and Methods

### 2.1. Database

We performed a 1-year (2018) retrospective cohort analysis of the Healthcare Cost and Utilization Project (HCUP) National Inpatient Sample (NIS). The NIS is the largest publicly available inpatient healthcare in the U.S.A. The NIS includes more than 7 million inpatient hospital records from 47 states and the District of Columbia, representing close to 97% of the U.S. population. The NIS contains information on all hospital stays. The large size of the NIS enables researchers and policymakers to identify, track, and analyze national trends in healthcare utilization, access, charges, quality, and outcomes. This study was exempt from institutional review board (IRB) approval because the NIS database contains deidentified patient data and involves minimal risk of patient health information (PHI) disclosure. This study was conducted in compliance with the ethical standards of the responsible institution on human subjects as well as with the Helsinki Declaration.

### 2.2. Study Design

We included all adult patients (age ≥ 18 years) presenting with a primary diagnosis of “MGUS” based on the International Classification of Diseases (ICD)-10th Revision-Clinical Modification diagnostic codes. We included those who had “MGUS” and “AFIB” based on the ICD-10th codes. In the NIS database, the first listed diagnosis is defined as the “condition established after study to be chiefly responsible for occasioning the admission of the patient to the hospital for care”. To prevent the bias associated with the hierarchy of discharge diagnosis the database provides the original value of the first listed diagnosis (I10_DX1), whether blank or coded, and is retained in the first position of the diagnosis vector. Starting at the first secondary diagnosis (I10_DX2), the diagnoses are shifted during HCUP processing to eliminate blank secondary diagnoses. For example, if I10_DX2 and I10_DX4 contain non-missing diagnoses and I10_DX3 is blank, then the value of I10_DX4 is shifted into I10_DX3. Secondary diagnoses are never shifted into the first listed position (I10_DX1). In our study, only I10_DX1 was utilized to capture “MGUS” hospitalizations. In our study, I10_DX2–I10_DX40 was utilized to capture “AFIB”. This was performed to ensure that MGUS was the condition chiefly responsible for hospitalization and to eliminate hospitalizations due to AFIB. We excluded patients who were less than 18 years of age, patients declared dead on arrival, and those diagnosed with “multiple myeloma”, “monoclonal gammopathy with renal significance”, or other types of malignancy. Patients with atrial flutter (I483, I484, I4892), sick sinus syndrome (I495), ventricular fibrillation (I4901), ventricular flutter (I4902), and those with other rhythm or conduction abnormalities (I440, I441, I442, I4430, I4439, I444, I445, I4460, I4469, I447, I450, I4510, I4519, I452, I453, I454, I455, I456, I4581, I4589, I459, I462, I468, I469, I470) were all excluded.

### 2.3. Patient Stratification

Patients were stratified into 2 groups based on the presence of AFIB. We have used the following ICD-10 diagnostic code (I10_DX1) to capture “MGUS”: (D472). We have used the following ICD-10 diagnostic codes (I10_DX2–I10_DX40) to capture “AFIB”: paroxysmal atrial fibrillation (I480), persistent atrial fibrillation (I481), chronic atrial fibrillation (I482), and unspecified atrial fibrillation (I4891).

### 2.4. Data Points

The following data points were recorded for each patient: patient demographics (age, sex, primary payer method, and household income), patient comorbidities (coronary artery disease (CAD), alcohol use disorder, chronic liver disease, diabetes mellitus (DM), chronic kidney disease (CKD), excessive weight loss, hypertension (HTN), chronic obstructive pulmonary disease, current cigarette smoking, stroke, obesity, dyslipidemia (DLD), heart failure (HF) and non-rheumatic valvular heart disease). We also analyzed data on admission [weekday and elective admissions].

### 2.5. Outcome Measures

The primary outcome measures were index in-hospital complications, mortality, hospital length of stay (LOS), hospital charges, and discharge disposition.

### 2.6. Statistical Analysis

We performed descriptive statistics. Continuous parametric variables were reported using a mean and standard deviation. Continuous nonparametric variables were reported using a median and interquartile range. Categorical variables were reported as counts and proportions. To compare the characteristics of the 2 groups, we used the independent *t*-test for continuous parametric variables and the Mann–Whitney U test for continuous nonparametric variables. The chi-square test was used to assess differences in categorical variables.

To ascertain the effect of AFIB on patient outcomes while adjusting for measurable confounding factors, we performed propensity score matching using a 1:1 matching ratio. The following variables were measured: demographics, admission characteristics, insurance status, and medical comorbidities. A logistic regression model was used to generate a propensity score (ranging from 0 to 1) for each patient. A nearest-neighbor model match, using a caliper width of 0.1, was performed to identify patients who were subsequently included in the post-match analysis. We considered a *p* value of less than 0.05 (*p* < 0.05) to be statistically significant. All statistical analyses were carried out using the Statistical Package for the Social Sciences (SPSS, version 26; SPSS, Inc., Chicago, IL, USA).

## 3. Results

### 3.1. Descriptive Analysis

#### Pre-Match Analysis

A total of 9007 patients with MGUS met the study’s inclusion criteria. The mean age of the entire patients with MGUS and AFIB was 78 ± 9 years and 58% were males. Non-elective admissions constituted 90% of hospitalizations. The most common primary expected payer was Medicare (87%) followed by private insurance (9%). Patients with a lower median household income defined as an income between $1–$58,999 constituted 45% of the cohort. The most common comorbidities that this population suffered from were HTN (56.7%), HF (57.4%) non-rheumatic heart disease (13.8%), and CAD (45.5%). Pre-match analysis of the baseline characteristics revealed significant differences between patients in the two groups in terms of demographics, primary expected payer, median household income, and comorbidities (Table 1).

### 3.2. Post-Match Analysis

A total of 2404 MGUS patients with AFIB (58.6% male; mean age 78 ± 9 years) included in this study during the year 2018 were matched for baseline characteristics with 2404 patients without AFIB (57.7% male, aged 78 ± 9 years) in the control group. A total of 9007 patients with MGUS met the study’s inclusion criteria. The mean age of the entire patients was 78 ± 9 years and 58% were males. Elective admissions constituted 77% of hospitalizations. The most common primary expected payer was Medicare (87%) followed by private insurance (9%). Patients with a lower median household income defined as an income between $1–$58,999 constituted 45% of the cohort. The most common comorbidities that this population suffered from were hypertension (56.7%), heart failure (57.4%), DLD (40.8%), diabetes mellitus (35.9%), obesity (17.3%), CKD (18.5%), and coronary artery disease (45.5%). Pre-match analysis of the baseline characteristics revealed significant differences between patients in the two groups in terms of demographics, primary expected payer, median household income, and comorbidities. No significant differences in any of the remaining reported variables were noted after propensity score matching (Table 2).

### 3.3. Outcomes

#### 3.3.1. In-Hospital Complications

Patients with MGUS who have AFIB had a significantly increased rate of AKI (31.5% vs. 27.5%; *p* = 0.002) and pericarditis (2% vs. 1.2%; *p* = 0.029) compared to patients without AFIB (Table 3). However, both groups had no significant difference in rates of MI (4.2% vs. 3.3%, *p* = 0.096), DVT (2% vs. 2.8%, *p* = 0.073), and PE (0.8% vs. 1%, *p* = 0.454).

#### 3.3.2. Mortality and Short-Term Outcomes

Patients with MGUS who have AIFB also had significantly higher in-hospital mortality (5.6% vs. 3%; *p* < 0.001) and median length of stay (5 vs. 4 days, *p* < 0.001) than patients without AFIB (Table 3).

#### 3.3.3. Financial Burden

The median cost of hospitalization for AFIB, $43,729 ($23,560–$87,572), was significantly higher than its matched counterpart, $41,169 ($22,323–$74,321) *p* < 0.001. It is also notable to mention that patients with AFIB were less likely to have a routine home discharge (33.8% vs. 39.2%; *p* < 0.001), whereas patients without AFIB were more likely to be discharged to other facilities (33.7% vs. 30.2%; *p* = 0.009) further increasing the financial expenses associated with having AFIB. There were similar rates of discharges to home with home health care (24% vs. 25.3%; *p* = 0.3).

## 4. Discussion

This study evaluates the impact of AFIB on hospitalized patients with monoclonal gammopathy of undetermined significance (MGUS). The results of the study showed that patients with both MGUS and AFIB were significantly older, more likely to be male and white, and had more medical comorbidities than patients with only MGUS. After adjusting for baseline characteristics and comorbidities, none of the matching variables showed significant differences. To our knowledge, this is the first study to examine the impact of AFIB on mortality and hospital outcomes among MGUS patients. The prevalence of AFIB in hospitalized MGUS patients is as high as 26%, and further studies targeted at studying screening for AFIB in MGUS population are warranted.

Shwartz et al. showed an association between MGUS and multiple cardiovascular diseases, including atrial fibrillation, pericarditis, heart failure, heart blocks, and aortic valve disease. This association was suggested to be related to paraprotein infiltration of the myocardium and subsequent myopathy [2]. A more malignant course of the disease with earlier comorbid end-organ involvement could be speculated in patients with concomitant AFIB and MGUS. In fact, this paraprotein infiltrative condition could be a cause of myocardial atrial dysfunction leading to AFIB, as suggested by Schwartz et al [2].

Monoclonal gammopathy of renal significance (MGRS) has been described in the literature as AKI in patients with monoclonal gammopathies [7]. In our study, we found a statistically significant difference between MGUS patients with AFIB and AKI. Multiple other circumstances can explain this finding. It could be hypothesized that similar to myocardial involvement, a similar mechanism could occur in the kidney and lead to MGRS if the extent of MGUS progresses with a component of AFIB. Therefore, routine evaluation for kidney involvement in patients with MGUS and AFIB, including monitoring for decreased renal function and proteinuria, is recommended.

While it is still uncertain whether the association between MGUS and cardiovascular risk factors is casual [4], Shwartz et al. demonstrated an increased prevalence of such factors in MGUS patients [2]. Chhabra et al. noted that the presence of AFIB in acute pericarditis is often related to the incidental discovery of a masked paroxysmal AFIB or the existence of underlying comorbidities that predispose patients to AFIB development (such as an enlarged left atrium) [8]. In our study, patients with both MGUS and AFIB exhibited a higher incidence of pericarditis compared to patients with MGUS alone, which may suggest infiltration in the pericardium and subsequent inflammation similar to the pathogenesis of AFIB. Additionally, they had a higher prevalence of non-rheumatic valvular disease, which may be explained by the latter being more common in patients with AFIB [9].

Kapoor et al. demonstrated an increased likelihood of MI in patients with MGUS [4]. However, in our study, there was no statistically significant association between patients with concurrent MGUS and AFIB and MI. Schwartz et al. indicated that MGUS is a chronic inflammatory state that leads to atherosclerosis and the involvement of inflammatory biomarkers [2]. Since we matched for CAD, it is possible that we found no significant difference in terms of MI rates. Additionally, some of those patients may be on antiplatelets and anticoagulants for prophylactic management, which could mask an increased association.

Our study found no significant difference in the risk of DVT and PE in patients with AFIB and MGUS compared to those with only MGUS. Interestingly, MGUS is known to be linked with an increased risk of DVT [10]. This could be attributed to the fact that this patient population is already on therapeutic anticoagulation, which could prevent the formation of these thromboembolic complications.

MGUS has been associated with an increased mortality rate [2], which is mainly attributed to cardiovascular disease as the leading cause of death in the elderly population [11]. Additionally, atrial fibrillation is known to increase the risk of mortality due to its cardiac-related implications, such as heart failure and an increased rate of comorbidities, as well as hematologic-related implications, such as venous thromboembolism (VTE) or pulmonary embolism (PE), and treatment-related implications, such as bleeding secondary to anticoagulation [12]. Our study found a higher rate of in-hospital mortality in patients with both MGUS and AFIB, which may be related to the impact of AFIB in these patients. This information could be useful for clinicians in managing these patients, including careful monitoring, which may have implications for their risk of progressing to MM. Moreover, given the higher prevalence of medical comorbidities in the MGUS and AFIB group, closer monitoring may be necessary, which may explain the higher in-hospital mortality rate, the longer median length of stay, and the increased financial burden seen in these patients. Our study also yielded similar findings.

Our study has several limitations that should be taken into consideration. Firstly, the use of the NIS database has inherent limitations, and the reliance on ICD-10 codes for identifying patient diagnoses may lead to potential coding errors, especially since MGUS is an asymptomatic condition and we are unable to assess how MGUS was the condition responsible for admission. Secondly, the absence of information on anticoagulation in the dataset limited our ability to evaluate the impact of anticoagulation on in-hospital mortality and complications. Thirdly, we were unable to stratify further into different categories of AFIB, and the inclusion of patients with valvular AFIB may have affected our results, despite efforts to include relevant covariates, such as heart failure and cardiomyopathy. Fourthly, the underreporting of MGUS in many hospitals may have led to a biased sample, and our results may not be representative of the true prevalence of the condition. Fifthly, we were unable to investigate the impact of different categories of immunoglobulins and the presence of cardiac amyloidosis, which is known to be associated with AFIB. AFIB was not defined as the admitting diagnosis in our study, but it may have still acted as an important confounder that may have contributed to increased rates of HF decompensation, stroke, ACS, AF ablation, and/or pacemaker implantation during hospitalization increasing LOS, medical costs, and mortality. ICD-10 diagnostic codes do not include patients with pacemaker rhythms, and those may have been undetected. Finally, it is important to note that this study was conducted at a single time point in time without follow-ups. Additionally, we are also limited by the absence of a control group that includes a non-MGUS counterpart; hence, cautious interpretation of the results should be taken into consideration.

Despite these limitations, our study has several strengths. We utilized a nationwide database that is representative of the general U.S. population and matched for important covariates that may have confounded our results.

## 5. Conclusions

Our study underscores the importance of accounting for AFIB as a potential confounding factor in MGUS patients as it can impact patient outcomes and affect the interpretation of results. Adjusting for relevant baseline characteristics and comorbidities, our study found that AFIB was associated with increased inpatient mortality, AKI, pericarditis, hospital charges, and length of stay among hospitalized MGUS patients. Cautious interpretation of the results is recommended; hence, further investigation is needed to fully comprehend the relationship between MGUS, AFIB, and patient outcomes and to establish the optimal management approach for these patients. Additionally, studies are necessary to explore the underlying mechanisms of AFIB in MGUS and its impact on the progression of MGUS to MM.

This knowledge may aid in guiding the clinical management of these patients, including the consideration of anticoagulants, which may influence their risk of MM progression. Given the higher prevalence of medical comorbidities in the MGUS and AFIB group, and the greater risk of complications, further research is needed to assess whether closer monitoring may be necessary in this patient population, especially accounting for the higher in-hospital mortality, median length of stay, and financial burden observed in our study.

## Figures and Tables

**Table 1:** Pre-Match Baseline Characteristics of Entire Study Sample.

Variable	MGUS & AFIB (n = 2404)	MGUS without AFIB (n = 6603)	*p* Value
Demographics			
Age, y, mean ± SD	78 ± 9	72 ± 12	<0.001
Sex, male, n (%)	1408 (58.6)	3399 (51.5)	<0.001
White, n (%)	1849 (76.9)	4331 (65.6)	<0.001
Black, n (%)	360 (15.0)	1471 (22.3)	<0.001
Admission Characteristics, n (%)			
Weekday Admission	1867 (77.7)	5245 (79.4)	0.068
Non-Elective Admission	2164 (90.0)	5678 (86.0)	<0.001
Primary expected payer, n (%)			
Medicare	2093 (87.1)	5077 (76.9)	<0.001
Private insurance	223 (9.3)	927 (14.0)	<0.001
Medicaid	60 (2.5)	417 (6.3)	<0.001
Median household income, $, n (%)			
1–45,999	517 (21.5)	1636 (24.8)	0.001
46,000–58,999	583 (24.3)	1555 (23.5)	0.489
59,000–78,999	643 (26.7)	1713 (25.9)	0.442
79,000+	661 (27.5)	1699 (25.7)	0.092
Medical Comorbidities, n (%)			
Coronary Artery Disease	1093 (45.5)	1827 (27.7)	<0.001
Alcohol Use Disorder	63 (2.6)	233 (3.5)	0.032
Chronic Liver Disease	217 (9.0)	546 (8.3)	0.253
Diabetes Mellitus	862 (35.9)	2478 (37.5)	0.146
Excessive Weight Loss	78 (3.2)	243 (3.7)	0.324
Hypertension	1362 (56.7)	2962 (44.9)	<0.001
Chronic Obstructive Pulmonary Disease	561 (23.3)	1138 (17.2)	<0.001
Current Smokers	130 (5.4)	625 (9.5)	<0.001
Stroke	222 (9.2)	526 (8.0)	0.054
Obesity	416 (17.3)	1051 (15.9)	0.115
Non-Rheumatic Valvular Heart Disease	332 (13.8)	427 (6.5)	<0.001
Heart Failure	1381 (57.4)	1827 (27.7)	<0.001
Dyslipidemia	980 (40.8)	2821 (42.7)	0.096
CKD	445 (18.5)	1221 (18.5)	0.983

AFIB: atrial fibrillation, CKD: chronic kidney disease, MGUS: monoclonal gammopathy of undetermined significance, $: dollar.

**Table 2:** Post-Match Baseline Characteristics of Entire Study Sample.

Variable	MGUS & AFIB (n = 2404)	MGUS without AFIB (n = 2404)	*p* Value
Demographics			
Age, y, mean ± SD	78 ± 9	78 ± 9	0.411
Sex, male, n (%)	1408 (58.6)	1387 (57.7)	0.559
White, n (%)	1849 (76.9)	1858 (77.3)	0.757
Black, n (%)	360 (15.0)	348 (14.5)	0.625
Admission Characteristics, n (%)			
Weekday Admission	1867 (77.7)	1852 (77.0)	0.605
Non-Elective Admission	2164 (90.0)	2147 (89.3)	0.421
Primary expected payer, n (%)			
Medicare	2093 (87.1)	2109 (87.7)	0.487
Private insurance	223 (9.3)	214 (8.9)	0.652
Medicaid	60 (2.5)	56 (2.3)	0.707
Median household income, $, n (%)			
1–45,999	517 (21.5)	525 (21.8)	0.779
46,000–58,999	583 (24.3)	591 (24.6)	0.788
59,000–78,999	643 (26.7)	644 (26.8)	0.974
79,000+	661 (27.5)	644 (26.8)	0.581
Medical Comorbidities, n (%)			
Coronary Artery Disease	1093 (45.5)	1086 (45.2)	0.839
Alcohol Use Disorder	63 (2.6)	65 (2.7)	0.858
Chronic Liver Disease	217 (9.0)	201 (8.4)	0.413
Diabetes Mellitus	862 (35.9)	870 (36.2)	0.810
Excessive Weight Loss	78 (3.2)	72 (3.0)	0.619
Hypertension	1362 (56.7)	1313 (54.6)	0.155
Chronic Obstructive Pulmonary Disease	561 (23.3)	545 (22.7)	0.583
Current Smokers	130 (5.4)	122 (5.1)	0.605
Stroke	222 (9.2)	230 (9.6)	0.693
Obesity	416 (17.3)	395 (16.4)	0.419
Non-Rheumatic Valvular Heart Disease	332 (13.8)	294 (12.2)	0.103
Heart Failure	1381 (57.4)	1369 (56.9)	0.855
Dyslipidemia	980 (40.8)	987(41.1)	0.894
CKD	445 (18.5)	431 (17.9)	0.664

AFIB: atrial fibrillation, CKD: chronic kidney disease, MGUS: monoclonal gammopathy of undetermined significance, $ dollar.

**Table 3:** Outcomes: a comparison of outcomes between two groups of patients, those with both MGUS and AFIB and those with only MGUS. The outcomes compared include the incidence of various in-hospital complications, such as acute kidney injury, myocardial infarction, deep vein thrombosis, pericarditis, and mortality.

Outcomes	MGUS & AFIB (n = 2404)	MGUS without AFIB (n = 2404)	*p* Value	Odds Ratio (OR) with Confidence Interval (CI)
In-Hospital Complications, n (%)				
Acute Kidney Injury	758 (31.5)	662 (27.5)	0.002	1.23 (95% CI: 1.08–1.41)
Myocardial Infarction	102 (4.2)	80 (3.3)	0.096	1.28 (95% CI: 0.94–1.74)
Deep Vein Thrombosis	48 (2.0)	67 (2.8)	0.073	0.71 (95% CI: 0.49–1.02)
Pulmonary Embolism	20 (0.8)	25 (1.0)	0.454	0.80 (95% CI: 0.44–1.44)
Pericarditis	48 (2.0)	29 (1.2)	0.029	1.69 (95% CI: 1.12–2.54)
Hospital Charges, $, median [IQR]	43,729 [23,560–87,572]	41,169 [22,323–74,321]	<0.001	1.08 (1.05–1.12)
Hospital Length of Stay, d, median [IQR]	5 [3–9]	4 [2–7]	<0.001	
Discharge Disposition, n (%)				
Routine to Home	812 (33.8)	943 (39.2)	<0.001	
Home Health Care	578 (24.0)	609 (25.3)	0.300	
Other Facility	809 (33.7)	725 (30.2)	0.009	
In-Hospital Mortality, n (%)	134 (5.6)	71 (3.0)	<0.001	1.97 (1.93, 5.52)

AFIB: atrial fibrillation, MGUS: monoclonal gammopathy of undetermined significance, $ dollar.

## Data Availability

The data that support the findings of this study are available from National Inpatient Sample (NIS).

## References

[1] Oganesyan A., Gregory A., Malard F., Ghahramanyan N., Mohty M., Kazandjian D., Mekinian A., Hakobyan Y. (2022). Monoclonal gammopathies of clinical significance (MGCS): In pursuit of optimal treatment. Front. Immunol..

[2] Schwartz B., Schou M., Ruberg F.L., Rucker D., Choi J., Siddiqi O., Monahan K., Køber L., Gislason G., Torp-Pedersen C. (2022). Cardiovascular Morbidity in Monoclonal Gammopathy of Undetermined Significance: A Danish Nationwide Study. JACC CardioOncol..

[3] Go R.S., Rajkumar S.V. (2018). How I manage monoclonal gammopathy of undetermined significance. Blood.

[4] Kapoor P., Rajkumar S.V. (2022). Cardiovascular Associations with Monoclonal Gammopathy of Undetermined Significance: Real or Coincidental?. JACC CardioOncol..

[5] Nesheiwat Z., Goyal A., Jagtap M. (2023). Atrial Fibrillation. StatPearls.

[6] Therneau T.M., Kyle R.A., Melton L.J., Larson D.R., Benson J.T., Colby C.L., Dispenzieri A., Kumar S., Katzmann J.A., Cerhan J.R. (2012). Incidence of monoclonal gammopathy of undetermined significance and estimation of duration before first clinical recognition. Mayo Clin. Proc..

[7] Menè P., Moioli A., Stoppacciaro A., Lai S., Festuccia F. (2021). Acute Kidney Injury in Monoclonal Gammopathies. J. Clin. Med..

[8] Chhabra L., Bhattad V.B., Sareen P., Khalid N., Spodick D.H. (2015). Atrial fibrillation in acute pericarditis: An overblown association. Heart.

[9] Thomas K.L., Jackson L.R., Shrader P., Ansell J., Fonarow G.C., Gersh B., Kowey P.R., Mahaffey K.W., Singer D.E., Thomas L. (2017). Prevalence, Characteristics, and Outcomes of Valvular Heart Disease in Patients with Atrial Fibrillation: Insights From the ORBIT-AF (Outcomes Registry for Better Informed Treatment for Atrial Fibrillation). J. Am. Heart Assoc..

[10] Kristinsson S.Y., Fears T.R., Gridley G., Turesson I., Mellqvist U.-H., Björkholm M., Landgren O. (2008). Deep vein thrombosis after monoclonal gammopathy of undetermined significance and multiple myeloma. Blood.

[11] Kristinsson S.Y., Björkholm M., Andersson T.M.-L., Eloranta S., Dickman P.W., Goldin L.R., Blimark C., Mellqvist U.-H., Wahlin A., Turesson I. (2009). Patterns of survival and causes of death following a diagnosis of monoclonal gammopathy of undetermined significance: A population-based study. Haematologica.

[12] Jackson I., Etuk A.S., Jackson N. (2022). Impact of Atrial Fibrillation on Inpatient Outcomes Among Hospitalized Patients with Multiple Myeloma. Cureus.

